# Characterization of the ^1^H-MRS Metabolite Spectra in Transgender Men with Gender Dysphoria and Cisgender People

**DOI:** 10.3390/jcm10122623

**Published:** 2021-06-14

**Authors:** Sarah Collet, Sourav Bhaduri, Meltem Kiyar, Guy T’Sjoen, Sven Mueller, Antonio Guillamon

**Affiliations:** 1 Department of Endocrinology, Ghent University Hospital, 9000 Ghent, Belgium; 2 Department of Experimental Clinical and Health Psychology, Ghent University, 9000 Ghent, Belgium; Sourav.Bhaduri@liverpool.ac.uk (S.B.); meltem.kiyar@ugent.be (M.K.); sven.mueller@ugent.be (S.M.); 3 Department of Endocrinology, Center for Sexology and Gender, Ghent University Hospital, 9000 Ghent, Belgium; Guy.TSjoen@ugent.be; 4 Department of Personality, Psychological Assessment and Treatment, University of Deusto, 48007 Bilbao, Spain; 5 Departamento de Psicobiología, Facultad de Psicología, Universidad Nacional de Educación a Distancia, 28040 Madrid, Spain; aguillamon@psi.uned.es

**Keywords:** transgender men, neuroimaging, magnetic resonance spectroscopy, sex differences, creatine, myo-inositol, N-acetyl-aspartate

## Abstract

Much research has been conducted on sexual differences of the human brain to determine whether and to what extent a brain gender exists. Consequently, a variety of studies using different neuroimaging techniques attempted to identify the existence of a brain phenotype in people with gender dysphoria (GD). However, to date, brain sexual differences at the metabolite level using magnetic resonance spectroscopy (^1^H-MRS) have not been explored in transgender people. In this study, 28 cisgender men (CM) and 34 cisgender women (CW) and 29 transgender men with GD (TMGD) underwent ^1^H-MRS at 3 Tesla MRI to characterize common brain metabolites. Specifically, levels of N–acetyl aspartate (NAA), choline (Cho), creatine (Cr), glutamate and glutamine (Glx), and myo-inositol + glycine (mI + Gly) were assessed in two brain regions, the amygdala-anterior hippocampus and the lateral parietal cortex. The results indicated a sex-assigned at birth pattern for Cho/Cr in the amygdala of TMGD. In the parietal cortex, a sex-assigned at birth and an intermediate pattern were found. Though assessed post-hoc, exploration of the age of onset of GD in TMGD demonstrated within-group differences in absolute NAA and relative Cho/Cr levels, suggestive for a possible developmental trend. While brain metabolite levels in TMGD resembled those of CW, some interesting findings, such as modulation of metabolite concentrations by age of onset of GD, warrant future inquiry.

## 1. Introduction

Transgender people experience an incongruence between the sex they were born with and their gender identity. Some individuals are born as female, but identify as male (i.e., trans men, TM). Others are born as male and identify as female (i.e., trans women, TW) [1]. Cisgender persons, comprising cis men (CM) and cis women (CW), do not experience gender incongruence. Considered as an umbrella term, ‘transgender’ can be used to describe a broad range of people whose gender identity differs from their sex assigned at birth. Not only does it include people identifying as the opposite of the sex they were born with, it also includes people who are not exclusively masculine or feminine, or even neither or both. In line with the goals of the World Professional Association for Transgender Health to de-psychopathologize being trans, the trans-related terminology has been constantly evolving in recent years [2,3]. In the ICD-10, gender dysphoria was previously subsumed under the category of “mental and behavioral disorders”, which undoubtedly contributed to the combined stigmatization that is still associated with trans and with psychiatric disorders. This has changed since the advent of the ICD-11, in which the more universal term gender incongruence was proposed with no reference to emotional stress. Moreover, the diagnosis was removed from the chapter “Mental Health Disorders” and relocated to the chapter “Conditions related to Sexual Health” [4]. Over the years, trans populations have been increasingly recognized in health care settings. Many, but not all, trans people may experience a discomfort, or even distress, caused by the discrepancy between experienced and assigned gender identity (i.e., gender dysphoria, GD). In addition, trans people may suffer not only from GD as a result of manifesting primary and secondary sex characteristics but also from the expected associated gender roles. In turn, this may induce psychological, physical, and social affliction as well as result in high rates of mood and anxiety disorders [5]. In order to align the physical appearance with their gender identity, trans persons may request gender affirming hormone therapy (GAHT). In addition, some undergo gender affirming surgery. It has been widely accepted that an interdisciplinary approach is needed toward health and well-being. The growing field of trans care and its resultant guidelines, partially summarized in the World Professional Association for Transgender Health Standards of Care edition 7, are an undisputable sign of this [6].

Sex differences in the human brain have been widely investigated [7]. Not only is there widespread evidence for differences between the sexes in brain morphometric characteristics such as total brain volume [7,8,9] and grey matter and white matter proportions [7,10], but brain function also appears to differ between sexes [11,12]. In general, sex differences take two patterns in the brain: either more male than female or more female than male. The words “masculine” and “feminine” are used to describe brain morphological or behavioural traits that are typical of the males or females of a species, respectively. Masculinization and feminization refer to any change that makes an individual more like typical males or females. Demasculinization and defeminization denote any change that makes an individual less like a typical male or female [13]. This depends upon the region of interest and the specific measurements being assessed. Whether a certain brain characteristic is more masculine or feminine has been the subject of many research efforts in recent years. However, despite an exponential increase of trans research in general, and neuroimaging in particular, the neurobiological underpinnings of being trans remain to be described [2]. Past studies in trans people aimed to define whether the brains of trans persons differ from those of cis persons and to what extent before starting with GAHT. Various findings with various MRI techniques have been reported [2,14,15]. Taken together, current MRI work in trans people before initiation of GAHT has shown many inconsistencies regarding (dis)similarities between the brain characteristics of trans persons and cis persons. Research has found the trans brain to resemble the sex assigned at birth [16,17,18], the gender identity [11,19,20,21], or even suggesting an intermediate phenotype situated between that of their sex assigned at birth and their gender identity, or showcasing a different pattern altogether [22,23,24,25,26]. Surprisingly lacking from current neuroimaging research in this cohort is the opportunity to utilize MRI to assess metabolite distribution in the brain.

Magnetic resonance spectroscopy (MRS) is a non-invasive method for visualizing selected metabolites. This is important as it provides information about the neurochemical substrates of brain function (and possibly brain structure) [27,28,29]. Associated metabolites that can be measured with MRS in the human brain include N-acetyl aspartate (NAA), creatine (Cr), choline (Cho), glutamate and glutamine (Glx), myo-inositol (mI), and glycine (Gly). Since the advent of MRI, quite a few MRS studies have tried to compare metabolite spectra in various brain regions between CM and CW but with heterogeneous results [30,31,32,33,34,35,36]. Interestingly, a more recent study [37] highlighted a role of menstrual cycle (and thus gonadal hormones) in creatine level fluctuations in the prefrontal cortex of CW, which may thus contribute to prior reports of sex differences in creatine levels. Creatine, besides its function as a transient intracellular storage of metabolic energy, has a role as an antioxidant [38], modulates GABA_A_ and NMDA receptors [39,40], and participates in the homeostasis of water content in cells working as an osmolyte [41]. Consistent with this work, a study with a reasonably large sample size documented sex differences, among others, in ratios involving total creatine in the parietal lobe [32]. This is interesting because resting-state MRI work [42,43] as well as limited structural work [17,25,44,45] has documented structural differences in the parietal lobe in trans vs. cis persons. Moreover, as alluded to above, because trans persons experience gender dysphoria/incongruence and minority stress [46,47,48,49] as well as anxiety and depression [50,51,52], another brain region that may be of interest is the amygdala, given its involvement in anxiety [53,54], depression [55], and stress-related disorders [56]. In this region, one study in participants exposed to stress indicated elevated creatine levels as well as increased levels of NAA and mI [57].

A recent study by Williamson et al. (2021) examined the relationship between cerebral metabolites, brain volume, and cognitive performance [58]. They found that cerebral metabolite concentrations are associated with cortical and subcortical volumes. Although the exact underlying physiological relationships to regional changes in structure remain unclear, this provided support in generating our study hypotheses. Based on a review of quantitative structural and functional MRI studies, a neurodevelopmental hypothesis on gender has been proposed in which TMGD present a brain phenotype with feminine, masculine, and defeminized traits depending on the MRI respective measure [13,43]. Therefore, this study aimed to gain insight into sex-typical ^1^H-MRS spectra in trans men with GD (TMGD) relative to CM and CW in two brain regions, i.e., the amygdala-anterior hippocampus and the lateral parietal cortex. The selection of these brain areas as regions of interest was motivated by (1) the role of the amygdala in emotion processing and its contribution to trans people experiencing frequent ostracism, social rejection, and stress; and (2) earlier structural MRI research that had been carried out in the parietal cortex of cis people [32] and an animal study investigating a female rat androgenization model [59]. This current study sought to find evidence for the rodent model by Perez-Laso et al. [59].

Cortical thickness in the parietal region in CW appears to be larger than in CM [17]. As TM and CW show a similar developmental pattern in the parietal cortex [13,17], and because we expect volumetric changes to be reflected in altered metabolite concentration [58], we hypothesized that no differences exist between CW and TMGD regarding absolute metabolite levels and relative metabolite ratios in the parietal cortex. In the amygdala however, no volumetric differences have been reported in TM with respect to cisgender people. Derived from this and considering this is the first study on the subject, we could not advance a specific hypothesis in this brain region and worked with a data driven approach. Overall, we hypothesized that TMGD, when compared to CM and CW, would show absolute and relative concentrations of metabolites respective of their feminine, masculine, or defeminized brain characteristics in these two regions of interest.

## 2. Experimental Section

### 2.1. Study Design and Cohort

Twenty-nine TMGD (mean age = 27.8 years ± 11.1 years), 28 CM (mean age = 26.3 years ± 5.8 years), and 34 CW (mean age = 30.2 years ± 8.9 years) participated as part of a larger longitudinal project, reviewed and approved by the Ethical Committee of Ghent University Hospital, Belgium. From February 2019 until November 2020, TMGD were included in the study protocol before the initiation of gender affirming hormone treatment (GAHT). During that same period of time, cis participants were asked to join the study via advertising on social media or by word of mouth. After receiving oral and written information about the study design, the participants provided written informed consent. Inclusion criteria for the trans cohort were hormone naive people aged 17 years or older and having a diagnosis of GD before initiating GAHT. The same age criterion applied for cis participants. Exclusion criteria for both cohorts were the presence of a medical history of psychiatric and/or neurologic diagnoses as assessed by anamnesis. In addition, the Mini-International Neuropsychiatric Interview [60] was carried out by a clinical psychologist, assessing and keeping track of undiagnosed psychiatric symptoms in participants. Sexual orientation was self-reported at first visit. For the purpose of creating a homogeneous study sample, only people mainly attracted to the opposite gender relative to their gender identity at the moment of their first visit in our clinic were included. Thus, an analysis of sexual orientation was therefore not possible. After completion of a standardized MRI safety check, acquisition was performed for all participants at the baseline visit (T0) and 6–12 months follow-up. Due to Covid-19 related delays, only T0 baseline data are presented in this paper.

### 2.2. Magnetic Resonance Spectroscopy

#### 2.2.1. Image Acquisition

**Figure 1:**
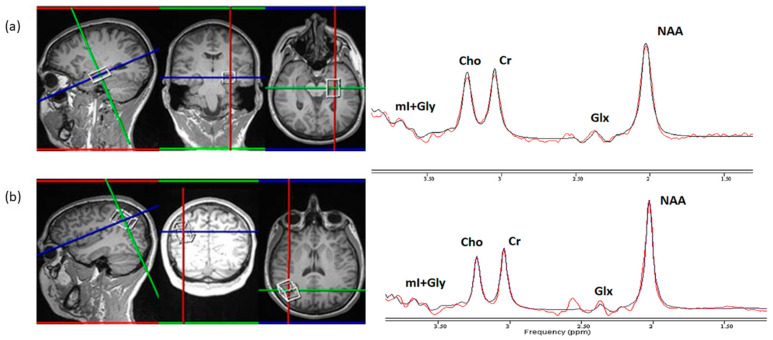
MRS voxel positioning. Left to right: sagittal view—coronal view—axial view—representative region-specific MR spectrum. mI + Gly = myo-inositol + glycine; Cho = choline; Cr = creatine; Glx = glutamate and glutamine, and NAA = N-acetyl aspartate. Images are in radiological orientation (L = R). (**a**) Voxel placement and generated ^1^H-MRS spectrum in the amygdala and the anterior hippocampus; (**b**) Voxel placement and generated ^1^H-MRS spectrum in the lateral parietal cortex.

Water suppressed and unsuppressed single voxel magnetic resonance spectroscopy (MRS) data were collected using point-resolved spectroscopy (PRESS) sequence in order to quantify the following brain metabolites: choline (Cho), creatine (Cr), N-acetyl aspartate (NAA), myo-inositol + Glycine (mI + Gly) and glutamate and glutamine (Glx) using a 3T Siemens Magnetom^®^ Prisma MRI scanner (Siemens Healthcare, Erlangen, Germany). A 64-channel receiver head coil was used for data acquisition. The following acquisition parameters were used: TE = 75 ms, TR = 3000 ms, bandwidth (BW) = 2000 Hz, no. of data points = 1024. No. of averages (Navg) were set to 128 and 8 for the water suppressed data and unsuppressed data, respectively. Using an intermediate TE, very close to the setting we have used, it had been shown previously that resonance at 2.35 parts per million overwhelmingly represents the glutamate resonance [61]. The FOV dimensions for the two regions of interest (ROI) were AP = 30 mm, HF = 20 mm, and RL = 20 mm (lateral parietal), and AP = 20 mm, HF = 15 mm, and RL = 20 mm (amygdala/anterior hippocampus). The FOV for the single voxel spectroscopy sequence was set to the above values in the scanner console to optimally fit the box to the respective ROIs (Figure 1). The voxel placement in the amygdala-anterior hippocampus was located in the left hemisphere. The left hippocampus was preferred because of the more important role in neuropsychiatric disorders [62]. For optimal signal-to-noise ratio, we placed a box within the confines of the amygdala including the anterior hippocampus as testing revealed unsatisfactory results with the amygdala, only. For the lateral parietal cortex, the voxel was placed in the right hemisphere, similar to a previous study investigating cortical thickness in TM and cisgender people [17]. CHESS [63] water suppression was performed to retrieve the water-suppressed data. T1 MPRAGE images (9° flip angle, TR/TE/TI 2300/2.96/900 ms, 192 slices, 1.0 mm thick, 256 × 256 matrix) were acquired prior to spectroscopy for the positioning of the spectroscopy voxel. Manual and automatic shimming were performed using Siemens software FASTMAP to maintain the full-width half-maximum (FWHM) of the water peak around 20 Hz [64].

#### 2.2.2. Image Processing

Proton spectra show a resonance of water that is 100,000 times higher than that of brain metabolites. It must be suppressed to enable evaluation of specific brain metabolites. Therefore, prior to performing data quantification on the water suppressed spectroscopy data, an HLSVD filter with model order of 25 was used to suppress the residual water peak at 4.7 ppm [65,66]. To increase the digital resolution of the data, apodization (with Lorentzian function and a factor of 2 Hz) and zero filling (by a factor of 2) were performed successively on the data. All these steps were performed using the jMRUI software [67]. The fitting of metabolite peaks was achieved using QUEST algorithm [68,69] within jMRUI. A basis set of desired metabolites was simulated using the tool NMR scope [70] inside jMRUI. The metabolite peaks, Cho (at 3.2 ppm), Cr (at 3.03 ppm), NAA (at 2.01 ppm), mI + Gly (at 3.56 ppm), and Glx (at 2.35 ppm and 3.74 ppm), were fitted. Cramér–Rao Lower Bound (CRLB) criteria was used to evaluate the quality of fitting. The CRLB/amplitude < 20% was selected as a criterion to discriminate well fitted metabolites from poorly fitted ones. Poorly fitted ones were excluded, resulting in different numbers of subjects per metabolite per region of interest. NAA/Cr, Cho/Cr, mI + Gly/Cr, Glx/Cr, and NAA/mI + Gly ratios were then calculated by taking individual metabolite amplitude ratios with respect to the amplitude of Cr or mI + Gly. In order to perform absolute quantification of metabolites, the water amplitude was calculated from the unsuppressed water data and used as an internal reference to estimate the absolute concentrations of the metabolites. The absolute concentrations of the metabolites were estimated using the following equation:

(1) $$C_{metabolite}=\left(\frac{2}{np}\right).\left(\frac{A_{metabolite}}{A_{water}}\right).\frac{\left(f_{GM\;}\times \;R_{water\_GM\;}\times \;C_{water\_GM}+f_{WM\;}\times \;R_{water\_WM}\times \;C_{water\_WM}+f_{CSF\;}\times \;R_{water\_CSF}\times \;C_{water\_CSF}\right)\;}{\left(1-f_{CSF}\right)\;\times \;R_{met}}\;$$

where:

(2) $$R_{water\_y}=exp\left(\frac{-TE}{T2_{water\_y}}\right)\left\{1-exp\left(\frac{-TR}{T1_{water\_y}}\right)\right\}$$

(3) $$R_{met}=exp\left(\frac{-TE}{T2_{met}}\right)\left\{1-exp\left(\frac{-TR}{T1_{met}}\right)\right\}$$

*A_metabolite_* is the area of the metabolite peak, *A_water_* is the area of the water peak calculated from the unsuppressed water data, *np* being the number of protons in a given metabolite, and *R_water_y_* is the relaxation correction factor for water signal in compartment y (GM for grey matter, WM for white matter, and CSF) with *T*1*_water_y_*, *T*2*_water_y_* being the relaxation times for water in compartment y with *T*1*_water_WM_* = 1084 msec, *T*2*_water_WM_* = 69 msec, *T*1*_water_GM_* = 1820 msec, *T*2*_water_GM_* = 99 msec, *T*1*_water_CSF_* = 4300 msec, and *T*2*_water_CSF_* = 503 msec [71,72,73], also known as the Stanisz values. *R_met_* is the relaxation correction factor for metabolites with *T*1*_met_*, *T*2*_met_* being the relaxation times of the metabolites obtained from literature [74,75]. The output variable *C_metabolite_* is metabolite concentration (mM). *C_water_WM_* = 36.1 mol/L, *C_water_GM_* = 43.3 mol/L, and *C_water_CSF_* = 53.8 mol/L are the water concentration values used as internal reference obtained from literature [76]. *f_GM_*, *f_WM_*, *f_CSF_* are the fractions of GM, WM, and CSF, respectively, calculated by segmentation of the co-registered MRS voxel on the T1 MPRAGE image using SPM12 toolbox in MATLAB as performed before in literature [77]. The Equation (1) used above gives an estimation of the concentration of the metabolites of interest in the GM and WM tissue fractions of the voxel [76].

### 2.3. Statistical Analysis

Count data on population characteristics (i.e., nicotine consumption, drug use, exercise, education level) were analyzed using the Chi square test. Clinical parameters such as age, BMI, and age of onset of GD were analyzed by using multivariate analyses of co-variance (MANCOVAs). Spectroscopic measures were log transformed to obtain normal distribution and were compared using MANCOVAs. Several prior studies have documented an influence of nicotine consumption and exercise on the MRS spectrum [78,79,80,81], which also differed among the present 3 groups. Therefore, all analyses used these two factors, along with age, as possible covariates of interest. By comparison, though education level and anxiety scores, which were assessed by the Mini-International Neuropsychiatric Interview carried out by a clinical psychologist, also differed between groups, they did not impact our results. The same MANCOVAs were run with anxiety and education scores included as covariates. Because these did not result in any change, they were not included. Moreover, as there were too few participants with high depression scores, we did not consider mood disorders as a covariate. All main effects of group analyses yielding significant *p* values were FDR corrected. All significant differences were further explored post hoc by running MANCOVAS using a Bonferroni correction on the significant main effects. The group differences in metabolite levels in terms of absolute amplitude concentrations of NAA, Cho, Cr, mI + Gly, and Glx were calculated. In addition, relative ratios of NAA/Cr, Cho/Cr, mI + Gly/Cr, Glx/Cr, and NAA/mI + Gly were also compared between groups. Alpha was set at *p* = 0.05 (two-tailed). Effect sizes were calculated using partial eta square. All analyses were conducted using SPSS Statistics 26 (IBM Corp., Armonk, NY, USA).

## 3. Results

Data on participant characteristics were available in 29 TMGD, 34 CW, and 28 CM. Gender identity groups did not differ regarding age or BMI (both *p* > 0.20). There were significantly more smokers in the TMGD group (42.9%) relative to the cis groups (X(2) = 11.45, *p* = 0.003). More than half of both CM and CW exercised regularly (*n* = 39). This was different from the TMGD (X(4) = 23.84, *p* < 0.001) where 16 out of 26 did not work out or engage in any physical activities. Drug use was defined as any scope of use of legal or illegal drugs without medical purposes and was similar in all groups (X(2) = 0.73, *p* = 0.695). The age of onset of GD in TMGD was self-reported at first visit and was categorized by stages of life in which the participants first experienced gender incongruent feelings. Twenty-seven TMGD were able to provide an answer; in 40.1% of them, this first occurred in childhood, for 37%, around pubertal age, and for 22.2%, in adult life. Educational attainment was assessed. TMGD appeared to be less educated than both cis groups (X(6) = 21.0, *p* = 0.002). Characteristics of the study population are shown in Table 1.

### 3.1. Amygdala and the Anterior Hippocampus

**Figure 2:**
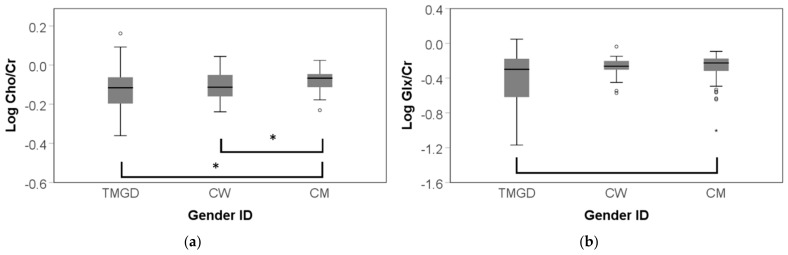
Group distributions of relative ratios in the amygdala-anterior hippocampus by gender identity (Gender ID). TMGD = trans men with GD; CW = cis women; CM = cis men; * *p* = <0.05; mild outliers are marked with a circle (°), extreme outliers are marked with an asterisk (*) (**a**) Log Cho/Cr was significantly higher in CM compared to TMGD and CW; (**b**) Log Glx/Cr approached, but did not reach, a significant *p* value.

Generated metabolite data for the amygdala and anterior hippocampal region were available from 23 TMGD, 29 CW, and 28 CM. Missing data resulted from poor quality MRS collection. No statistically significant group differences emerged regarding log transformed absolute metabolite amplitudes of NAA, Cho, Cr, mI + Gly, and Glx (all *p* > 0.05). However, a main group effect emerged for the relative ratio for Cho/Cr (F(2, 85) = 7.910, *p* = 0.002 FDR corrected, η^2^ _p_ = 0.176). Post hoc Bonferroni corrected tests revealed higher Cho/Cr ratios in CM versus TMGD (mean = 0.072, SD = 0.022, *p* = 0.004) and in CM versus CW (mean = 0.064, SD = 0.018, *p* = 0.002) (Figure 2). Although the Glx/Cr ratio only approached significance (F(2, 82)= 3.128, *p* = 0.050, η^2^ _p_ = 0.078), a significant effect was found when comparing CM to TMGD post hoc whilst Bonferroni correcting (mean = 0.123, SD = 0.049, *p* = 0.045). Similar mean spectroscopic measures were found across all categories of gender identity for NAA/Cr, NAA/mI + Gly, and mI + Gly/Cr (all *p* > 0.24).

### 3.2. Lateral Parietal Cortex

**Figure 3:**
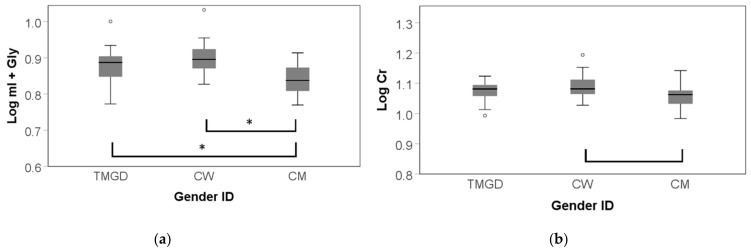
Group distributions of absolute metabolite concentrations in the lateral parietal cortex by gender identity (fender GD). TMGD = trans men with GD; CW = cis women; CM = cis men; * *p* = <0.05; mild outliers are marked with a circle (°). (**a**) Log mI + Gly was significantly lower in CM compared to TMGD and CW; (**b**) Log Cr was nearly significantly higher in CW compared to CM.

Data for the lateral parietal cortex were available from 24 TMGD, 27 CW and 23 CM. Multivariate analysis of the log transformed absolute concentration of metabolites and post hoc Bonferroni corrected tests indicated a higher log mI + Gly in both TMGD (mean = 0.043, SD = 0.014, *p* = 0.012) and CW (mean = 0.050, SD = 0.012, *p* < 0.001) when compared to CM (F(2, 75) = 8.590, *p* = 0.004 FDR corrected, η^2^ _p_ = 0.202). A near statistically significant effect between Cr levels across gender identities was also found (F(2, 77) = 3.056, *p* = 0.054, η^2^ _p_ = 0.082) where only cis groups differed after post hoc Bonferroni correction (mean = 0.026, SD = 0.011, *p* = 0.048), resulting in higher Cr concentrations in CW relative to CM (Figure 3). Mean NAA, Glx, and Cho concentrations were similar in all groups (all *p* > 0.26).

**Figure 4:**
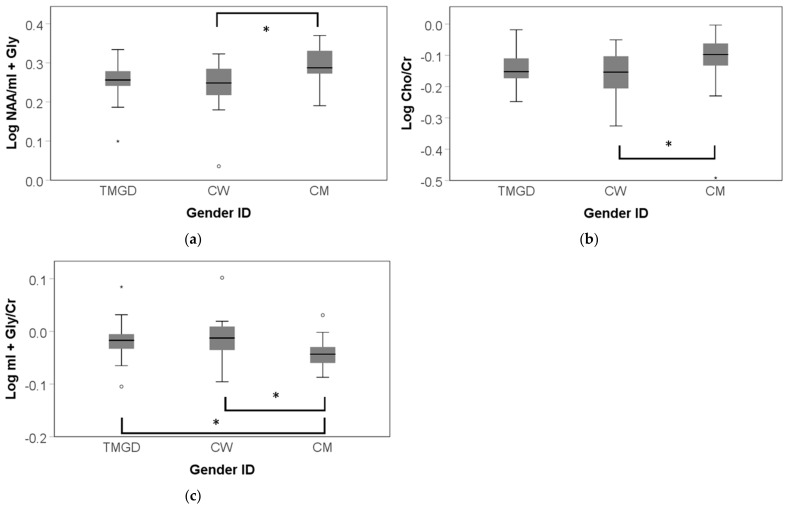
Group distributions of relative ratios in the lateral parietal cortex by gender identity (gender ID). TMGD = trans men with GD; CW = cis women; CM = cis men; * *p* = <0.05; mild outliers are marked with a circle (°), extreme outliers are marked with an asterisk (*). (a) Log NAA/mI + Gly was significantly higher in CM compared to CW; (**b)** Log Cho/Cr was significantly higher in CM compared to CW; (**c**) Log mI + Gly/Cr was significantly lower in CM compared to CW and TMGD.

Of the log transformed relative ratios, NAA/mL + Gly (F(2, 81) = 4.823, *p* = 0.015 FDR corrected, η^2^ _p_ =0.124) and Cho/Cr (F(2, 82) = 5.027, *p* = 0.015 FDR corrected, η^2^ _p_ = 0.129) differed significantly among the groups (Figure 4). Overall, post hoc Bonferroni analyses demonstrated higher ratios in CM versus CW (NAA/mI + Gly: mean = 0.042, SD = 0.014, *p* = 0.009; Cho/Cr: mean = 0.058, SD = 0.019, *p* = 0.008).

The sole exception to this pattern was mI + Gly/Cr (F(2, 80) = 4.428, *p* = 0.016 FDR corrected, η^2^ _p_ =0.115) for which after post hoc Bonferroni correction, CM appeared to have lower ratios compared to CW (mean = -0.024, SD = 0.009, *p* = 0.026) and TMGD (mean = −0.026, SD = 0.010, *p* = 0.041). NAA/Cr (F(2, 82) = 2.832, *p* = 0.066, η^2^ _p_ = 0.077) did not reach the set alpha level after correction for age, and Glx/Cr was similar across the three groups (*p* = 0.201). No statistically significant differences were observed between TMGD and CM or CW.

### 3.3. Post Hoc Analyses of Age of Onset of GD

**Figure 5:**
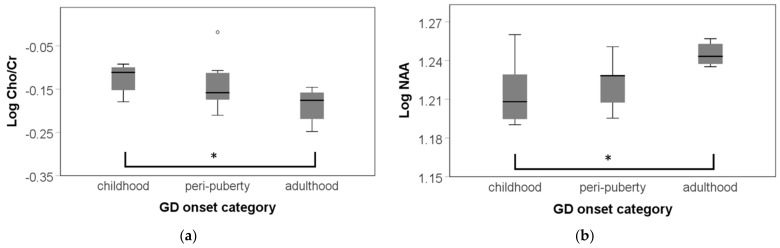
Group differences in TMGD in function of age of onset of gender dysphoria (GD) in the lateral parietal cortex. GD onset categories were defined as childhood, peri-puberty, and adulthood. * *p* = <0.05; mild outliers are marked with a circle (°). (**a**) Absolute NAA levels by age of onset. Log NAA was significantly lower in TMGD first experiencing GD in childhood compared to those first experiencing GD in adulthood. (**b**) Relative Cho/Cr ratio by age of onset. Log Cho/Cr was significantly higher in TMGD first experiencing GD in childhood relative to those first experiencing GD in adulthood.

In addition, because no studies have to date documented group differences in trans people based on age of onset of GD, we sought to examine exploratively and post-hoc whether such differences existed. An age of onset of GD effect emerged in absolute NAA levels and Cho/Cr ratios in the lateral parietal cortex (Figure 5). Only testing metabolite levels corrected for age within TMGD depending on GD age of onset, post hoc Bonferroni corrected tests revealed lower NAA absolute amplitudes in TMGD who first experienced GD in childhood (mean = −0.03, SD = 0.011, *p* = 0.027) compared to those first experiencing GD in adulthood (F(2, 22) = 4.206, *p* = 0.045 FDR corrected, η^2^ _p_ = 0.286). In contrast, Cho/Cr ratios were found to be higher in the TMGD first experiencing GD in childhood compared to those first experiencing GD in adulthood (mean = 0.061, SD = 0.023, *p* = 0.049) (F(2, 23) = 3.617, *p* = 0.045 *p* = 0.045 FDR corrected, η^2^ _p_ = 0.256).

## 4. Discussion

This study sought to characterize, by virtue of MRS, the levels of five common brain metabolites in two regions of interest, the amygdala-anterior hippocampus and the lateral parietal cortex of CM, CW, and TMGD. Based on a review of structural and functional MRI studies and the developmental hypothesis of GD [13,43], we had hypothesized that, compared to CM and CW, TMGD would show the concentration of metabolites respective of their feminine, masculine, or defeminized brain characteristics in these two regions of interest. As such, we hypothesized that no differences exist between CW and TMGD regarding absolute metabolite levels and relative metabolite ratios in the parietal cortex. In the amygdala however, we did not advance a specific hypothesis and used a data driven approach. Three main findings emerged. The first appears to be a central role of creatine, which seemed to drive the effects found in this study as seen through altered ratios involving this metabolite. Second, while main effects of absolute levels were also found in the parietal cortex, this was not the case for the amygdala/anterior hippocampus region. Third, a post-hoc finding was that age of onset of GD appeared to influence NAA levels and Cho/Cr ratio in the parietal lobe.

As predicted, Cr appeared to play a central role in the findings, particularly in ratios involving Cr. Sex differences in Cr absolute concentration in the parietal lobe were marginally significantly higher in CW than in CM. Previous work had indicated such an effect in the frontal lobe [82], whereas a longitudinal study found that Cr levels in the prefrontal cortex fluctuate in CW while they are stable in CM [37]. However, others found higher Cr concentrations in CM in the right frontal lobe and a greater age-related decrease of Cr in basal ganglia in CM compared to CW [30]. Besides different methodologies used to measure Cr, it has been suggested that Cr is asymmetrically distributed across the hemispheres in CM and CW [32,37] and that could explain between-studies inconsistencies.

Despite these inconsistencies in the literature, we found sex differences to be similar as reported by Maudsley et al. [32] for relative Cho/Cr ratios in the parietal cortex. As such, in our study, CM compared to CW had greater relative ratios for Cho/Cr, while TMGD did not differ from either of the other two genders. However, in the amygdala, TMGD, like CW, had lower trending relative Glx/Cr ratios than CM, indicating a pattern consistent with sex assigned at birth. In addition to the modulation of findings by Cr, analyses of metabolite distribution in the parietal lobe also revealed group differences with respect to mI + Gly. In the lateral parietal cortex, CW and TMGD had greater content of mI + Gly than CM. As a result, this difference affected the NAA/mI + Gly ratio of these two genders with respect to CM. Consistent with the creatine findings, mI + Gly concentration and NAA/mI + Gly ratio patterns in the parietal lobe also suggested a sex assigned at birth effect in TMGD. As a major organic osmolyte, mI regulates cell volume in response to osmotic stress [83]. It is also an astrocyte marker and the precursor of the phosphatidylinositol second messenger system [84]. Curiously, Zubiaurre-Elorza et al. reported structural MRI findings demonstrating a thicker parietal lobe cortex in CW and TM when compared to CM [17]. One hypothesis for greater mI + Gly in CW and TMGD could be that water content in astrocytes is important to maintain this structural difference. Therefore, one highly interesting conjecture might be that the documented structural gender differences in the parietal lobe may have some foundation in brain metabolite distribution [85].

In addition to findings in the parietal lobe, the selection of which was motivated by earlier structural MRI research in trans people, the amygdala-anterior hippocampus ROI had been chosen based on the notion that trans people experience frequent ostracism, social rejection, and stress. Given the involvement of the amygdala in emotion regulation [86], stress coping strategies [87], and social exclusion [88], one aim was to characterize metabolite ratios in this region in trans individuals. In the amygdala, TMGD also differ from CM with Cho and trending Glx ratios in respect to creatine. In both, TMGD show lower ratios than CM. This is the same profile we described in the parietal lobe. TMGD show a feminine pattern in relation to these ratios. Although scarce, existing MRS work in human and animal models has provided complementary findings to date. Whereas mice exposed to chronic psychosocial stress exhibited an increase in inositol levels in the amygdala [89], chronic mild physical stress (such as stroboscopic light) in rats resulted in elevated NAA levels in the ventral hippocampus [90]. However, due to genetic differences, reconciling findings between rats and mice [91], let alone rodents and humans [92], is not straightforward and thus complicates the picture. In the only human study we are aware of, patients with PTSD exhibited an elevated Cr and mI concentration in the left and right amygdala, respectively [57]. These rather limited data do not reconcile with the current findings. First, somewhat unexpected, sex differences in metabolites were not as prominent as in the parietal lobe region. Interestingly, while older neuroimaging work had postulated volumetric differences between CM and CW in the amygdala [7,93], more recent meta-analyses cast doubt on this supposed dimorphism [94]. Secondly, even though trans people are often exposed to a variety of social stressors, including stigma and discrimination [46,47,49], this did not appear to play a role in the current study. This is interesting, because the rather limited neuroimaging work on affective function in other brain regions in trans people has not yet assessed the involvement of ostracism in the existence of a possible trans brain phenotype [2,12,95,96].

In addition to characterizing metabolite spectra in TMGD, a particularly novel finding was that we were able to examine the potential influence of the time frame in which gender incongruence was first experienced on brain characteristics. To our knowledge no previous neuroimaging studies have investigated such effects. Two previous reports have documented age of onset of gender dysphoria symptoms in subgroups of trans people. A Dutch study from 2005 investigated the existence of developmental subtypes in groups at the time described as homosexual and non-homosexual trans persons, referring to the erotic attraction to one’s biological sex. Although no difference was found in the intensity of gender dysphoria between groups, the homosexual trans persons reported more symptoms of gender dysphoria in childhood than the non-homosexual trans persons [97].

In a pan-European study, Nieder and colleagues [98] documented that the majority of TM were more likely to have an early vs. late onset, whilst onset for TW was more balanced. In our data, this pattern was replicated with ~77% experiencing onset in childhood or peri-puberty. Moreover, TMGD first experiencing GD in childhood exhibited significantly lower NAA absolute amplitudes and higher Cho/Cr ratios in the lateral parietal cortex relative to TMGD with onset in adulthood. Although this analysis was performed post-hoc and characterized by a very small sample size (11 vs. 10 vs. 6 TMGD, respectively), the findings provide long-needed support for a neurodevelopmental hypothesis on potential subgroup differences within trans people [13]. Thus, future neuroimaging work should consider adding age of onset information to the analyses. Such inclusion would allow aligning the time course of gender development to brain development.

The present findings may also bear some clinical implications. As noted, no previous brain imaging studies have characterized the MRS spectrum in trans people. Moreover, previous studies focusing on gender differences between CM and CW have documented mixed findings [32,33,34,35,36,37], possibly depending on the investigated brain region. The current research extends these prior findings. Baseline characterization of essential brain metabolites in trans people may serve as a first step toward understanding the potential impact that gender-affirming hormone treatment may have on brain health in this cohort. Such a first step appears necessary before embarking on studies that examine how sex steroid hormones impact various brain metabolites.

Several limitations should be noted. Although previous studies have documented an effect of menstrual cycle on MRS data [37], unfortunately, we did not have any information on the menstrual cycle phase of participants. Therefore, the present findings have to be handled with caution in light of this limitation. However, given that it is probable that variation of menstrual cycle phase in TMGD and CW may have been random and cancelled each other out (within groups), and given that both groups resembled each other, and differed from CM, a potential moderating effect of menstrual cycle phase on the current data seems unlikely to change the main overall effect between groups. Nonetheless, future work will need to describe this issue in more detail. Secondly, it was not possible to discriminate the anterior hippocampus from the amygdala in the spectroscopic voxel upon image acquisition. As differences in metabolite levels between gender exist in the hippocampus [36], the contribution of this structure in our findings should be considered. Lastly, although the effect of age of onset of gender dysphoria on the metabolite spectrum was small, it was statistically significant and provides encouragement for future exploration. These findings should thus be considered preliminary but promising.

## 5. Conclusions

In conclusion, this ^1^H-MRS study demonstrates that in terms of main metabolites, TMGD may follow a pattern more consistent with sex assigned at birth in the amygdala but may show an intermediate phenotype for the lateral parietal cortex. Our data suggest the existence of a possible confounding effect of sex in magnetic resonance spectroscopy.

## Figures and Tables

**Table 1:** Baseline characteristics and distribution of smoking, exercise, drug use, and GD onset in the study population.

	TMGD (*n* = 29*)*	CW (*n* = 34*)*	CM (*n* = 28)
Mean age (years)	27.8 ± 11.9	30.2 ± 8.9	26.3 ± 5.8
Mean body mass index (kg/m^2^)	25.4 ± 6.2	23.4 ± 3.8	23.6 ± 4.3
Smoking			
Never	16 (57.1%)	32 (91.4%)	25 (83.3%)
Current	12 (42.9%)	3 (8.6%)	5 (16.7%)
Exercise			
None	16 (61.5%)	8 (22.9%)	4 (13.8%)
Occasionally	7 (26.9%)	9 (25.7%)	4 (13.8%)
Regularly	3 (11.5%)	18 (51.4%)	21 (72.4%)
Education			
Primary school degree	1 (3.4%)	0	0
High school degree	20 (69%)	10 (28.6%)	11 (36.7%)
Graduate school degree	8 (27.6%)	14 (40%)	7 (23.3%)
Postgraduate degree	0	11 (31.4%)	12 (40%)
Drug use			
Never	27 (93.1%)	34 (97.1%)	29 (96.7%)
Current	2 (6.9%)	1 (2.9%)	1 (3.3%)
Onset of GD		/	/
Childhood	11 (40.1%)		
Peri-puberty	10 (37%)		
Adulthood	6 (22.2%)		

## Data Availability

The data presented in this study are available on request from the corresponding author. The data are not publicly available because they contain information that could compromise research participant privacy.
